# Utility of therapeutic drug monitoring in the treatment of major depressive disorder

**DOI:** 10.1097/YCO.0000000000001046

**Published:** 2025-11-05

**Authors:** Georgios Schoretsanitis, Michael Paulzen

**Affiliations:** a Department of Psychiatry, Psychotherapy and Psychosomatics, Hospital of Psychiatry, University of Zurich, Zurich, Switzerland; b The Zucker Hillside Hospital, Psychiatry Research, Northwell Health, Glen Oaks, New York, USA and Department of Psychiatry at the Donald and Barbara Zucker School of Medicine at Northwell/Hofstra, Hempstead, New York, USA; c Alexianer Centre for Mental Health Aachen/Gangelt; d Department of Psychiatry, Psychotherapy and Psychosomatics, RWTH Aachen University, and JARA – Translational Brain Medicine, Aachen, Germany

**Keywords:** antidepressants, major depressive disorder, personalized treatment, pharmacokinetics, therapeutic drug monitoring

## Abstract

**Purpose of the narrative review:**

Response and remission rates in patients with major depressive disorder (MDD) treated with antidepressants are frequently not satisfying. Therapeutic drug monitoring (TDM), i.e. the quantification of antidepressant drug levels in blood and dose adjustment, is a modern and useful tool of personalized pharmacological treatment of MDD.

**Recent findings:**

Emerging evidence suggests that the use of TDM for antidepressants can be helpful in numerous clinical scenarios. Such scenarios include lack of treatment response, relapse, or adverse drug reactions related to antidepressants. The use of TDM is also indicated in specific patient subgroups, such as children, adolescents, pregnant women, elderly patients and patients with intellectual disabilities. Patients with polypharmacy and/or physical comorbidities may also benefit from TDM-guided antidepressant treatment. We critically reviewed TDM literature on antidepressants, summarizing therapeutic reference ranges and laboratory alert levels for antidepressants, although TDM is not equally recommended/supported for all antidepressants.

**Summary:**

The utilization of TDM as a tool for treatment optimization in clinical routine with antidepressants for patients with MDD offers a valuable method to improve safety and effectiveness. This work summarizes essential TDM knowledge for antidepressants and encourages the application of TDM as part of the clinical decision-making process.

## INTRODUCTION

The measurement of drug concentrations in blood (plasma or serum), known as therapeutic drug monitoring (TDM), provides valuable insight in psychopharmacotherapy regardless of the addressed mental disorder [1^▪▪^,^2^^▪▪^]. Specifically, the quantification of drug concentrations allows tailoring the therapeutic regimen, taking into consideration the patients’ individual characteristics and their particular impact on pharmacokinetic patterns [2^▪▪^]. The use of precision medicine tools like TDM in the psychopharmacological treatment of patients with major depressive disorder (MDD) may enhance efficacy, thereby improving response or remission rates and tolerability of antidepressants [3].

Efficacy and tolerability of antidepressants are major challenges in clinical routine; in one of the largest available naturalistic studies, one out of three patients with MDD did not respond to a first-line antidepressant treatment [4]. Apart from the burden of nonresponsiveness, up to 75% of patients may develop at least one antidepressant-related adverse drug reaction (ADRs) [5]. Using switches from a first-line pharmacological treatment of MDD with escitalopram to a different antidepressant as a surrogate of treatment failure in the largest available TDM cohort, authors found supra- and subtherapeutic concentrations more frequently in “switchers” compared to patients remaining under escitalopram [6^▪^]. This finding was replicated for escitalopram and sertraline in a more recently studied cohort [7], consolidating the hypothesis that patients with sub- and supratherapeutic concentrations more frequently suffer from poor treatment outcome and tolerability, highlighting the need for TDM as part of clinical routine in the treatment with antidepressants.

A number of further benefits arise when clinicians use TDM for antidepressants; for instance, antidepressant-related ADRs may be associated with lower rates of adherence to antidepressants [8,9]; the impact of nonadherence to antidepressants on treatment outcomes has been previously underscored including poor psychosocial outcomes, as well as increased suicide rates [10]. Apart from healthcare utilization and related costs [11,12], poor treatment outcomes related to nonadherence to antidepressants may also account for the considerable percentage of patients not achieving full remission of depressive symptoms in clinical routine [13]. Additionally, nonadherence to antidepressants may, at least partially, explain high relapse rates shortly after response or even remission following antidepressant treatment in the index episode [14]. In fact, the use of TDM to assess adherence to psychotropic agents, including antidepressants, holds a longstanding tradition in clinical routine [1^▪▪^].

Nevertheless, several MDD guidelines consider TDM only for a treatment with lithium [15], whereas other guidelines suggest TDM in case of lacking response for standard antidepressant doses [16]. Apart from these indications, TDM may be particularly valuable in the management of diverse specific MDD patient subgroups; some typical cases include the off-label prescription of antidepressants in subgroups, such as children and adolescents [17], women during pregnancy and lactation [18^▪^,^19^], elderly patients, and patients with intellectual disabilities [2^▪▪^]. Additional TDM indications include suspected drug-drug interactions [20] and somatic comorbidities [2^▪▪^], particularly in patients with polypharmacy, which reflects a common treatment strategy [21]. A full list of TDM indications in clinical routine has been previously published [2^▪▪^].

The TDM task force of the Association of Neuropsychopharmacology and Pharmacopsychiatry (AGNP) publishes consensus papers on the theoretical framework and clinical knowledge for the TDM use in clinical routine [2^▪▪^,^22^,^23^], which we highly suggest for clinicians interested in TDM. 

**Box 1:**
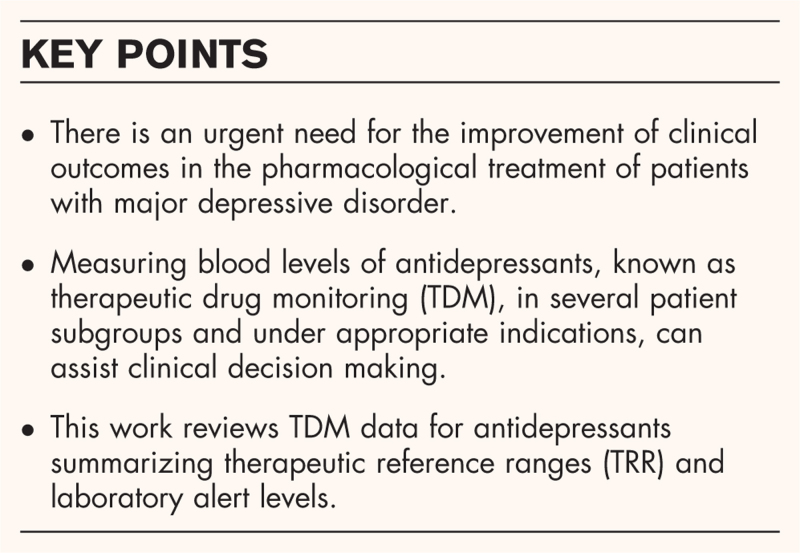
no caption available

## THERAPEUTIC DRUG MONITORING FOR ANTIDEPRESSANTS

Several fundamental notions for an appropriate use of TDM in clinical routine for patients with MDD should be addressed. We mainly focus on antidepressants, which are the mainstay of pharmacological treatment of MDD. However, several guidelines recommend lithium as an augmentation strategy [15,16]; TDM is part of lithium monitoring protocol in various countries [15,16,24]. Related knowledge mainly derives from studies including patients with bipolar disorder and is summarized elsewhere [2^▪▪^,^25^]. When lithium is used in augmentation strategies with antidepressants, a wider range of lithium levels, including values below 0.4 mmol/L has been reported in MDD responders (0.3–1.2 mmol/l) [26].

Apart from lithium, there are some additional medications frequently prescribed in augmentation to antidepressants, including antiseizure medications and antipsychotics. Nevertheless, as available TDM data for these medications mainly refer to their main indication (other than MDD), it is hard to extrapolate TDM knowledge for the main licensed indication to the prescription of such augmentation strategies in patients with MDD.

### Steady-state and sampling time

In the field of TDM, the term “steady-state” concentration describes the time point when the rate of medication input equals the rate of elimination. The time to reach steady-state is approximately 4–5 half-life times of the medication [2^▪▪^]; thus, for the majority of antidepressants, steady-state is expected to be reached within one week of maintenance dosing with the exception of fluoxetine and vortioxetine, which have a long half-life time (Table 1) [27,28]. The appropriate sampling time is right before the next antidepressant dose, referred to as the so-called trough level. In the unusual case of antidepressants prescribed at bedtime, the blood draw interval is 12 h.

**Table 1:** Therapeutic reference ranges and laboratory alert levels (in ng/ml) and elimination half-lives (*t*_1/2_) of antidepressants (modified from Hiemke *et al.* [2^▪▪^]) except for duloxetine, escitalopram and venlafaxine [32–34]

Drugs and active metabolites	Therapeutic reference ranges in blood (ng/ml)	Laboratory alert level (ng/ml)	*t*_1/2_ (h^a^)
Antidepressants for which TDM is strongly recommended			
Amitriptyline plus nortriptyline	80–200	300	10–28 18–44
Citalopram	50–110	220	38–48
Clomipramine plus N-desmethyl-clomipramine	230–450	450	16–60 37–43
Imipramine plus desipramine	175–300	300	11–25 15–18
Nortriptyline	70–170	300	18–44
Antidepressants for which TDM is recommended			
Bupropion Hydroxybupropion	10–100 850–1500	2000	1–15 17–47
Desipramine	100–300	300	15–18
Dothiepin	45–100	200	18–21
Doxepin plus nordoxepin	50–150	300	15–20
Duloxetine	20–120	240	9–19
Escitalopram	20–40	160	27–32
Fluvoxamine	60–230	500	21–43
Maprotiline	75–130	220	20–58
Milnacipran	100–150	300	5–8
Mirtazapine	30–80	160	20–40
Sertraline	10–150	300	22–36
Trazodone	700–1000	1200	4–11
Trimipramine	150–300	600	23–24
Venlafaxine plus *O*-desmethylvenlafaxine	140–600	800	14–18 10–17
Vortioxetine	10–40	80	57–66
Antidepressants for which TDM is useful			
Desvenlafaxine	85–380	450	10–17
Fluoxetine plus norfluoxetine	120–500	1000	4–6^a^ 4–16^a^
Levomilnacipran	80–120	200	6–9
Mianserin	15–70	140	14–33
Moclobemide	300–1000	2000	2–7
Paroxetine	20–65	120	12–44
Reboxetine	60–350	700	13–30
Tianeptin	30–80	160	2.5–3
Vilazodone	15–45	90	18–32
Antidepressants, for which TDM is potentially useful			
Agomelatine	7–300	600	1–2
Tranylcypromin	≤50	100	1–3

TDM, therapeutic drug monitoring; *t*_1/2_: elimination half-life.

a Elimination half-lives are provided in hours except for fluoxetine and norfluoxetine where they are provided in days.

### Levels of recommendations

Primarily for the older (i.e. tricyclic) antidepressants, so-called therapeutic reference ranges (TRR) have been established based on their association with clinical response in trials with sufficient reliability. The TRR is an essential target range for TDM guided pharmacotherapy. Its estimation requires determination of a lower and an upper limit of effective and tolerable drug concentrations in blood. Whenever a TRR is sufficiently reliable, TDM can be strongly recommended in clinical routine. Nevertheless, specificity and sensitivity vary not only between antidepressants, but also between trials. Furthermore, disentangling placebo and drug-specific effects can also perplex the investigation of the association between clinical effects and antidepressant levels [29]. Clarity is provided by the results of positron emission tomography (PET) including data from in vivo occupancy of serotonin transporters that can be very helpful when assessing thresholds of clinical effects [30].

For the tricyclic antidepressants amitriptyline, clomipramine, imipramine and nortriptyline, TDM is highly recommended for titration to target dose, as well as for special indications (Table 1). Specifically, antidepressant levels within the TRR are linked to better response, remission and relapse prevention, as well as tolerability; furthermore, TDM is also highly recommended for citalopram, for which data from one trial suggested that citalopram levels above 53 ng/ml at day 14 had a highly predictive value regarding treatment response [31].

For a second group of antidepressants, including bupropion, desipramine, dothiepin, doxepin, duloxetine, escitalopram, fluvoxamine, maprotiline, mirtazapine, sertraline, trazodone, trimipramine, venlafaxine and vortioxetine, TDM is recommended with a lower level of clinical confidence (recommendation level 2); this is because data linking level ranges to either clinical response or tolerability issues are less robust [2^▪▪^]. However, TDM may still help to increase the probability of response or remission in nonresponders, in that subtherapeutic antidepressant levels may be associated with risk of poor response (also known as pseudo treatment resistance).

For a third group of antidepressants, including desvenlafaxine, fluoxetine, levomilnacipran, mianserin, moclobemide, reboxetine, tianeptine and vilazodone, evidence is less supportive for TDM use (recommendation level 3), as the link between antidepressant levels and clinical effects has not been addressed sufficiently yet or only in a retrospective fashion; Nonetheless, TDM may be still be useful, but more evidence is needed to recommend TDM.

Lastly, for agomelatine and tranylcypromine, there is a lack of evidence associating clinical effects and blood levels (recommendation level 4), so that TDM is only considered as potentially useful.

However, regardless of the level of TDM recommendation for each antidepressant, there are some clinical scenarios where TDM can be valuable; for instance, in a patient not responding or experiencing an MDD relapse, the absence of a detectable antidepressant blood level is a crucial information, which can clearly support the clinical decision-making.

### Therapeutic reference ranges and laboratory alert levels

The therapeutic reference range (TRR) for antidepressants blood levels describe a level range consisting of a lower limit, below which clinical response is relatively unlikely, and an upper limit, above which ADRs are more frequent, or above which it is relatively unlikely that patients will further respond; TRRs from the last update of the AGNP TDM consensus guidelines [22] are provided in Table 1, except for duloxetine, escitalopram, venlafaxine and vilazodone, where TRRs have been recently revised [32–34].

Clinicians need to bear in mind that TRRs represent an orienting, population-based tool, which may not always apply to every patient. For instance, some patients might respond even at subtherapeutic antidepressant levels, whereas, some patients may only respond at supratherapeutic levels without reporting tolerability problems. Hence, the clinical value of regular TDM can be seen in the fact that it helps enabling clinicians to identify the individual's therapeutic range thereby helping to tailorize treatment strategies. Within a measurement-based framework obtaining several antidepressant blood levels once the patient responds without tolerability problems, clinicians can define the individual's unique therapeutic range. This range is particularly valuable for the long-term treatment, when adherence issues, drug-drug interactions, intercurrent diseases, or changes in liver or renal functioning may occur; TRRs for antidepressants refer to the parent compound, except for amitriptyline, clomipramine, doxepin, fluoxetine, imipramine, and venlafaxine, where active moiety concentrations are determined; the active moiety, AM, represents the sum of the parent compound and the active metabolite, which is pharmacologically active for the antidepressants mentioned above. Additionally, TDM for bupropion may introduce a challenge due to the instability of the parent compound, so that various laboratories do not offer measurement of bupropion levels, unless the blood sample is immediately frozen after collection [35]; If a lab does not offer the quantification of bupropion, it is a common practice to measure hydroxybupropion levels [35].

Note that TRRs apply to the indication of MDD. Nevertheless, antidepressants are increasingly prescribed for different indications apart from MDD [36]. However, evidence for diagnosis-specific TDM is limited. Therefore, here we explicitly focus on MDD-specific TRRs. Together with the TRRs, we provide laboratory alert levels (Table 1), that practically represent threshold levels, above which the risk for ADRs may exponentially increase. Antidepressant levels above the laboratory alert levels should be immediately reported by the laboratory to the prescribing clinician, who may consider a therapeutic regimen adjustment in presence of ADRs. If an individual patient tolerates the medication well, clinicians will need to assure the correctness of sampling time, i.e. check that the blood level was drawn at 24 h after the last dose, check that the patient is taking the antidepressant as prescribed, rule out overdose, rule out drug-drug interactions due to co-medications (including medications from other disciplines and over-the-counter medications) that can inhibit the metabolism of antidepressants, and monitor clinical signs implying an overdose/excessive blood levels before acting accordingly. This process actually describes how to apply TDM in general. It is important to remember that an isolated antidepressant level should not define any clinical decision. Apart from the role of co-medication(s), contextual information is required to interpret antidepressant levels. This includes demographic and clinical characteristics, such as age, sex, smoking habits and the indication for the TDM request. Needless to say that the clinical signs (symptom severity, response or remission, and ADRs related to antidepressants) are central when interpreting antidepressant levels. In addition, we encourage multiple measurements of antidepressant levels as part of a comprehensive baseline profiling, but also as part for a more reliable problem solving process, for which TDM was initially requested.

### Limitations and future hopes

Despite the potential of TDM as a measurement-based tool to improve the effectiveness of antidepressants in clinical routine, there are several inherent limitations; TRRs refer to population-based data and, thus, may not apply to every individual. In fact, it is not uncommon in clinical practice to treat patients, who may pharmacokinetically behave as “outliers”. Such outliers include patients with psychiatric and/or physical comorbidities, multiple co-medications and may suffer from more severe/chronic forms of MDD. Thus, one might expect that such patient subgroups might respond to different antidepressant blood level ranges. Of note, the pharmaceutical industry has routinely included pharmacokinetic assessments in their antidepressant trials at least for the last two decades. However, we are not aware of any published therapeutic antidepressant blood range for none of the recently approved antidepressants. Since these data exist, we urge the pharmaceutical companies to either publish correlational data between antidepressant blood levels and clinical response and safety/tolerability parameters, or to make these data publicly available. The further analysis of such data will help to overcome methodological deficits of current evidence, such as pooling data from samples with patients at different MDD phases (e.g., acute vs. maintenance treatment). Moreover, antidepressant blood level ranges usually derive from patients with MDD, although antidepressants are frequently prescribed in other diagnostic groups. More TDM data should be collected in additionally approved and/or off-label indications for which antidepressants are routinely used. Besides that, data are still limited for many antidepressants, hampering a solid investigation of the relationship between blood level ranges and clinical response and tolerability. Unfortunately, this data scarcity may contribute to the limited use of TDM in clinical routine. A typical example for the urgent need for more data refers to different age groups, such as the elderly, children and adolescents. Currently, TRRs for antidepressants in children and adolescents are extrapolated from adult cohorts [37]. Recent pooled analyses of TDM data for antidepressants in pediatric samples have overcome limitations of underpowered samples [17]. The increasing availability of TDM data may gradually leverage the determination of age-specific TRRs in pediatric patients, which, in turn, could stimulate the implementation of TDM in the prescription of antidepressants in pediatric populations. Additionally, the use of TDM heavily depends on the availability of laboratories that can analyze the blood level of any or specific antidepressant. Finally, costs arguably introduce a strong barrier to TDM; on the other hand, the use of TDM for antidepressants may reduce the duration of inpatient treatment thereby reducing inpatient treatment related costs [38^▪^]. Data highlighting the cost-effectiveness of TDM for antidepressants is currently limited; we call for the collection of such important data that will help policymakers in the future to support this important tool of precision medicine. Despite limitations, data indicate that TDM is a valuable, so far underutilized measurement-based tool of precision medicine that requires more application and deserves more attention.

## CONCLUSION

Despite the unanimously embraced call for personalized therapy of MDD, currently available tools in clinical routine are limited. In the cross-indication use of antidepressants, TDM represents a unique method that quantifies interindividual pharmacokinetic variability to enhance effectiveness and safety in the treatment with antidepressants [39]. The tradition of TDM in the prescription of antidepressants dates back in 1985, when the first attempt to establish guidelines was undertaken from the American Psychiatric Association [1^▪▪^]. Three decades later, the implementation of TDM into clinical practice has progressed only to a limited extent [40,41], although the potential of TDM is widely embraced. Apart from the so much needed additional research, TDM implementation as part of antidepressant prescription may depend on its consideration in MDD guidelines and educational curricula. For instance, the European Psychiatric Association has published a psychopharmacology-pharmacotherapy curriculum for postgraduate training with an extensive section on TDM in psychiatry [42]. Ultimately, it remains desirable that a broader implementation of TDM in the pharmacological MDD treatment patient may help to further improve treatment outcomes.

## Acknowledgements

*None.*

### Financial support and sponsorship

*None.*

### Conflicts of interest

*G.S. has served as a consultant for Dexcel, HLS Therapeutics, Lundbeck, Saladax and Thermo Fisher and has received speaker's fees from HLS Therapeutics, Lundbeck and Saladax. MP has received speaker's fees from Johnson & Johnson, ROVI, Neuraxpharm, Idorsia, Teva, Lundbeck and Otsuka. He has served as a consultant for Johnson & Johnson, Otsuka, Idorsia and ROVI. He is editor of the internet-based drug–drug interaction program PSIAC (“http://www.psiac.de”)*.

## REFERENCES AND RECOMMENDED READING

Papers of particular interest, published within the annual period of review, have been highlighted as:

▪ of special interest
▪▪ of outstanding interest
